# Silver linings: how COVID‐19 expedited differentiated service delivery for HIV

**DOI:** 10.1002/jia2.25807

**Published:** 2021-10-28

**Authors:** Anna Grimsrud, Peter Ehrenkranz, Izukanji Sikazwe

**Affiliations:** ^1^ HIV Programmes and Advocacy International AIDS Society Cape Town South Africa; ^2^ Global Health Bill & Melinda Gates Foundation Seattle Washington; ^3^ Centre for Infectious Disease Research in Zambia (CIDRZ) Lusaka Zambia

**Keywords:** community, COVID‐19, differentiated service delivery, HIV, multi‐month dispensing, self‐care, services, virtual medicine

“In the rush to return to normal, use this time to consider which parts of normal are worth rushing back to.”

– Dave Hollis, author

As we write this Editorial at the beginning of July 2021, the World Health Organization (WHO) has reported that the African continent is experiencing its worst week of the COVID‐19 pandemic and that COVID‐19 cases are rising in all six of the WHO's global regions [1, 2]. There is a serious imbalance in global distribution of COVID‐19 vaccines with only 1% of people in Africa and low‐income countries being fully vaccinated and 85% of doses globally having been administered in high‐ and upper‐middle‐income countries [1, 3]. Amid this recent surge of infections and the inequities in access to vaccines, it is increasingly clear that the cycles of COVID‐19 waves are likely to continue until the virus that causes COVID‐19 transitions from pandemic to endemic [4].

Africa is home to the largest number of people living with HIV – two‐thirds of the 38 million people living with HIV reside in on the continent, with 20.7 million people living with HIV in East and southern Africa alone [5]. Many sub‐Saharan African countries have experienced an increased strain on their existing health infrastructure, including on human resources for health, since the onset of the COVID‐19 pandemic, prompting countries to move swiftly to ensure uninterrupted supply of antiretroviral therapy (ART) and limit visits to health facilities for people on ART [6]. As a result, many of the previous barriers to the implementation of differentiated service delivery (DSD) models for HIV treatment, such as a recent documented undetectable viral load [7, 8], were unlocked, at least temporarily. With the rapid adaptation of national policies on HIV delivery to the realities of COVID‐19 restrictions and lockdowns, eligibility for entry in DSD for HIV treatment models was expanded, longer refills of ART were prescribed and dispensed, virtual models of care were innovated, and the role of community models for HIV treatment delivery was reinforced. Additionally, ways to expand DSD services beyond treatment to encompass HIV testing and prevention services were explored.

In November 2020, we published a call for abstracts for both quantitative and qualitative data on the impact of these adaptations with the intention of drawing attention to the changes made to DSD for HIV in response to COVID‐19. Evidence was needed to understand the effect of the temporary measures being implemented on health outcomes among people living with HIV.

Since our call, data have emerged that alleviates some of the initial concern that COVID‐19 would lead to interruptions in HIV treatment delivery and result in considerable increases in morbidity, mortality and HIV incidence [9]. The November 2020 UNAIDS global AIDS update emphasized that while COVID‐19 had led to decreases in access to HIV prevention, testing and consequently ART initiation, the number of people on ART has continued to increase with 27.5 million people on treatment worldwide at the end of 2020 [10]. Similarly, data from the Kwa‐Zulu Natal (KZN) province in South Africa found a 48% decrease in HIV testing and 46% reduction in new ART initiations but no marked change in the number of ART collection visits during South Africa's first lockdown (April‐July 2020) [11].

In March 2021, WHO launched updated recommendations on service delivery that expand eligibility criteria for access to DSD for HIV treatment, re‐validate recommendations to extend ART refills and clinic visits for those who are established on ART, promote integration of family planning and non‐communicable disease management within HIV programmes and encourage tracing and re‐engagement for those in a treatment interruption [12]. These guidelines are based on data collected before COVID‐19, and therefore, more recent evidence will be critical to inform future updates for DSD that go beyond HIV treatment.

This supplement includes 11 articles from the more than 50 submissions received and addresses many of the areas we highlighted as being of particular interest. In this editorial, we summarize six key themes that emerge from this supplement and what this new data add to our understanding of accelerating access to DSD for HIV before scoping the future for DSD.

**1. Virtual support on mobile phones can accelerate ART initiation and facilitate monitoring in facilities and communities**

In response to COVID‐19, many digital platforms were utilized to reduce in‐person delivery of services [13]. Data from Amatavete et al. in Thailand outline how telehealth follow‐up after same‐day ART initiation [14] led to positive outcomes in terms of referral to long‐term treatment facilities and retention among those receiving phone follow‐up compared to in‐person follow‐up. Virtual follow‐up via telehealth was also part of the DSD adaptations in the United States Veterans Health Administration (VA) programme described below [15]

**2. DSD for HIV treatment can benefit those recently started on ART and those on second‐line regimens**

Two articles within the supplement found non‐inferior HIV‐related health outcomes among populations previously unable to access DSD for HIV treatment. Pooling data from two trials in Lesotho and Zimbabwe, Fatti et al. found that those who were referred to out‐of‐facility DSD models for HIV treatment after only 6–12 months on ART had similar outcomes as compared to those who had been on treatment for a year or longer at the time of referral [16]. Further, this study found that those who had annual clinical visits had at least non‐inferior outcomes compared to those with more frequent (every 3 months) in‐person clinical consultations. This is important evidence, given that the updated WHO guidance still recommends clinic visits every 3–6 months [12]. Novel evidence on the outcomes of people on second‐line ART within DSD for HIV treatment models is described in an article from KZN, South Africa [17]. No differences in retention or viral suppression were observed comparing clients on second‐line ART referred to community treatment distribution models versus those remaining in clinic‐based care.

**3. Extended ART refill durations should be a new standard of care**

Perhaps the most consistent innovation recommended for HIV services during COVID‐19 has been to increase access to and duration of multi‐month dispensing (MMD) of ART refills [18, 19, 20]. Using routinely collected data from 17 PEPFAR supported countries, Bailey et al. describe how the proportion of clients receiving 6 months of ART increased from 9% in April to June 2020 to 16% in the following quarter (July‐September 2020) [21]. MMD uptake was also expanded among important specific populations, including for children less than 15 years of age, for whom 3‐month dispensing of ART increased from 34 to 50% in the same period. An analysis of routine data from Zambia by Jo et al. provides additional nuance to the multi‐country PEPFAR results [22]. While participation in any DSD treatment model increased in 2020, there were significant obstacles in terms of choice of model and in access to longer dispensing intervals related to challenges in the supply chain.

**4. COVID‐19 has emphasized the importance of expanding access to community‐based services**

Data from the scale‐up of DSD for HIV treatment in Nigeria describes enrolment numbers and client health outcomes between 2018–2020 [23]. Of five models that were implemented, more than half of all clients referred participated in a community ART refill club (53%) and the largest increase in enrolment corresponded with the first COVID‐19 wave in Nigeria. In India, Pollard et al. facilitated discussions with gay men and other men who have sex with men, female sex workers and transgender women in two provinces to identify preferences for delivery of HIV prevention, testing and treatment services [24]. Community‐based approaches that are flexible were identified as critical for HIV prevention, testing and treatment services for the key populations interviewed in this study. In Botswana, two individual out‐of‐facility models for ART refills were explored: home delivery and collection from private pharmacies [25]. A pilot of home delivery through a courier service found that 84% of clients accepted home delivery with 91% of ART refills successfully delivered. Both prospective users and private pharmacies were approached in assessing the feasibility of ART refills from private pharmacies; 61% of the prospective users indicated interest and willingness to pay approximately USD$4/refill for two refills per year.

**5.  DSD for HIV is also relevant in more highly resourced settings**

Data from the United States VA highlight adaptations made in the United States parallel to those seen in less resourced contexts [15]. Adaptations included an increase in virtual follow‐up and the duration of ART refills. In 2020, virtual visits (predominantly by telephone) increased to 68% compared to 27% in 2019 and 50% of ART refills were for 3‐month ART refills or longer compared to 38% in 2019. Along with other published data from HIV providers in San Francisco [13], this VA data supports the acceptability of using virtual means to provide HIV services.

**6.  DSD is applicable for HIV prevention and tuberculosis treatment**

While much of DSD has historically focused on differentiating HIV treatment for those established on treatment, COVID‐19 has also accelerated adaptations to other parts of the HIV care continuum including prevention. In Zimbabwe, Matambanadzo et al. present data on how demand for pre‐exposure prophylaxis (PrEP) among sex workers increased during COVID‐19 lockdowns and was overcome through home delivery, extended PrEP dispensing and support via WhatsApp through providing mobile data and airtime [26]. Further, DSD must not be limited to HIV or HIV treatment alone. In the one commentary in the supplement, Tran et al. argue for the expansion of DSD for HIV treatment models to include tuberculosis treatment [27]. We agree—and while policy provisions were made in some countries to align HIV services and the delivery of other health commodities like family planning or medications for non‐communicable diseases, very little data on the implementation of these policies are available [6, 28].

## REMAINING GAPS IN THE EVIDENCE AND SCOPING THE FUTURE OF DSD FOR HIV AND BEYOND

Many of the remaining gaps require data from implementation science, such as the paucity of evidence available on the integration of HIV services with other disease areas [29]. Further analyses of routine data on how COVID‐19 adaptions impacted outcomes are also encouraged, including the effect of earlier access to MMD, particularly from ART initiation as well as for specific populations namely children and adolescents, those who are pregnant and breastfeeding and migrant populations. More data would also be welcome describing the perspectives and experiences of both recipients of care and healthcare providers of COVID‐19 adaptations to HIV service delivery. In addition, costing and financing data is missing—particularly relevant in making the argument for adaptations to be sustained.

In summary, the articles in this supplement contribute to a growing evidence base showing that modifications made in response to COVID‐19 should not be temporary, but rather part of a better service delivery system going forward that meets the needs of recipients of care. Indeed, COVID‐19 has quickened acknowledgement across diverse stakeholders that DSD is not just for people who are established on ART. Rather, COVID‐19 has presented an opportunity for a shift toward scale up of self‐care models in general—not just for HIV, but also for tuberculosis, chronic diseases like hypertension and diabetes, and routinely provided services like family planning. The key components of DSD for HIV treatment of reducing the number of clinical visits, separating them from a decreased frequency of drug dispensing and prioritizing out‐of‐facility models apply to all of these purposes. Constraints within many supply chains were compounded by COVID‐19 but should not prevent wider implementation of MMD or DSD models in general.

Highlighted in this supplement are the important roles of community‐based services and virtual platforms (telephone, SMS and videoconferencing) in decreasing barriers for accessing critical aspects of the clinical visits as well as the resources required to provide it. These shifts may indeed represent a silver lining to the pandemic—a renewed focus on leveraging improvements in health systems, including supply chain and information technology, to provide high quality person‐centred care.

## COMPETING INTERESTS

AG is a Deputy Editor of the Journal of the International AIDS Society. PE is an employee of the Bill & Melinda Gates Foundation.

## AUTHORS’ CONTRIBUTIONS

All authors were involved in the conceptualization of the article. AG wrote the first draft. All authors approved the final submission.

## FUNDING

AG's efforts was funded by the Bill & Melinda Gates Foundation INV002610. PE is an employee of the Gates Foundation. IZ is an employee of CIDRZ, with funding from PEPFAR, Bill & Melinda Gates Foundation and the NIH.
